# Increased body fat percentage in patients with hepatitis B and C virus infection

**DOI:** 10.1371/journal.pone.0200164

**Published:** 2018-07-02

**Authors:** Yuan-Yuei Chen, Wen-Hui Fang, Chung-Ching Wang, Tung-Wei Kao, Yaw-Wen Chang, Hui-Fang Yang, Chen-Jung Wu, Yu-Shan Sun, Wei-Liang Chen

**Affiliations:** 1 Department of Internal Medicine, Tri-Service General Hospital Songshan Branch, Taipei, Taiwan, Republic of China; 2 Division of Family Medicine, Department of Family and Community Medicine, Tri-Service General Hospital; and School of Medicine, National Defense Medical Center, Taipei, Taiwan, Republic of China; 3 Division of Geriatric Medicine, Department of Family and Community Medicine, Tri-Service General Hospital; and School of Medicine, National Defense Medical Center, Taipei, Taiwan, Republic of China; 4 Graduate Institute of Clinical Medical, College of Medicine, National Taiwan University, Taipei, Taiwan, Republic of China; 5 Division of Family Medicine, Department of Community Medicine, Taoyuan Armed Forces General Hospital, Taoyuan, Taiwan, Republic of China; Policlinico Universitario Campus Bio-Medico, ITALY

## Abstract

Accumulated evidence has suggested associations between glucose abnormalities and insulin resistance with hepatitis C virus (HCV) and hepatitis B virus (HBV) infections. However, few studies have reported the effect of hepatitis virus infections on body composition. Our aim was to explore the association of hepatitis virus infections with percent body fat (PBF) in a cross-sectional analysis. A total of 69226 subjects obtained from the health examinations at Tri-Service General Hospital (TSGH) from 2010 to 2016 were enrolled in the study. Participants were divided into subgroups based on the presence of hepatitis B surface antigen (HBsAg) and anti-HCV. PBF was measured by bioelectrical impedance analysis (BIA). A multivariable linear regression model was applied to test the association of hepatitis virus infections with PBF and glycemic status. In male participants, hepatitis virus infections were closely associated with increased PBF, especially in those subjects with HCV/HBV coinfection. HCV/HBV coinfection was positively correlated with fasting plasma glucose and postprandial glucose while HCV and HBV mono-infection were not. The impact of hepatitis virus infection on increased PBF was observed in general population with gender difference. A further study on the treatment of hepatitis virus infection might help prevent the development of obesity-related diseases.

## Introduction

Hepatitis virus infection is a progressive disease leading to the development of cirrhosis and even hepatocellular carcinoma (HCC) in approximately 20–30% of patients worldwide[1, 2]. Hepatitis B virus (HBV) and hepatitis C virus (HCV) infections were the two major etiologies of liver diseases that caused medical health and socioeconomic problems in Taiwan[3, 4]. Numerous studies have reported that hepatitis virus infection was related to the risk of type 2 diabetes mellitus (DM) and a higher percentage of patients with viral liver diseases had DM than did those with other pathogenesis[5, 6].

Insulin resistance is a pathological condition in which cells) fail to respond normally to insulin; it is responsible for cardiometabolic disorders[7]. Common features in obesity and DM are reduced insulin-stimulated glucose transport and metabolism in adipocytes and impaired suppression of hepatic glucose output[8]. Adipocytes are among the most highly insulin-responsive cell types[9]. Previous studies have suggested that increased adiposity was a principal contributor to insulin resistance[10].

The association between hepatitis virus infection and insulin resistance has been published previously[11, 12]. To our knowledge, no previous studies have explored the interaction between hepatitis virus infections and adipose tissue. Our aim was to analyze whether hepatitis virus infections including HCV, HBV and HCV/HBV coinfection would impact percent body fat (PBF) in the general population composed of participants from the health examinations at Tri-Service General Hospital (TSGH).

## Methods

### Study design

**Fig 1:**
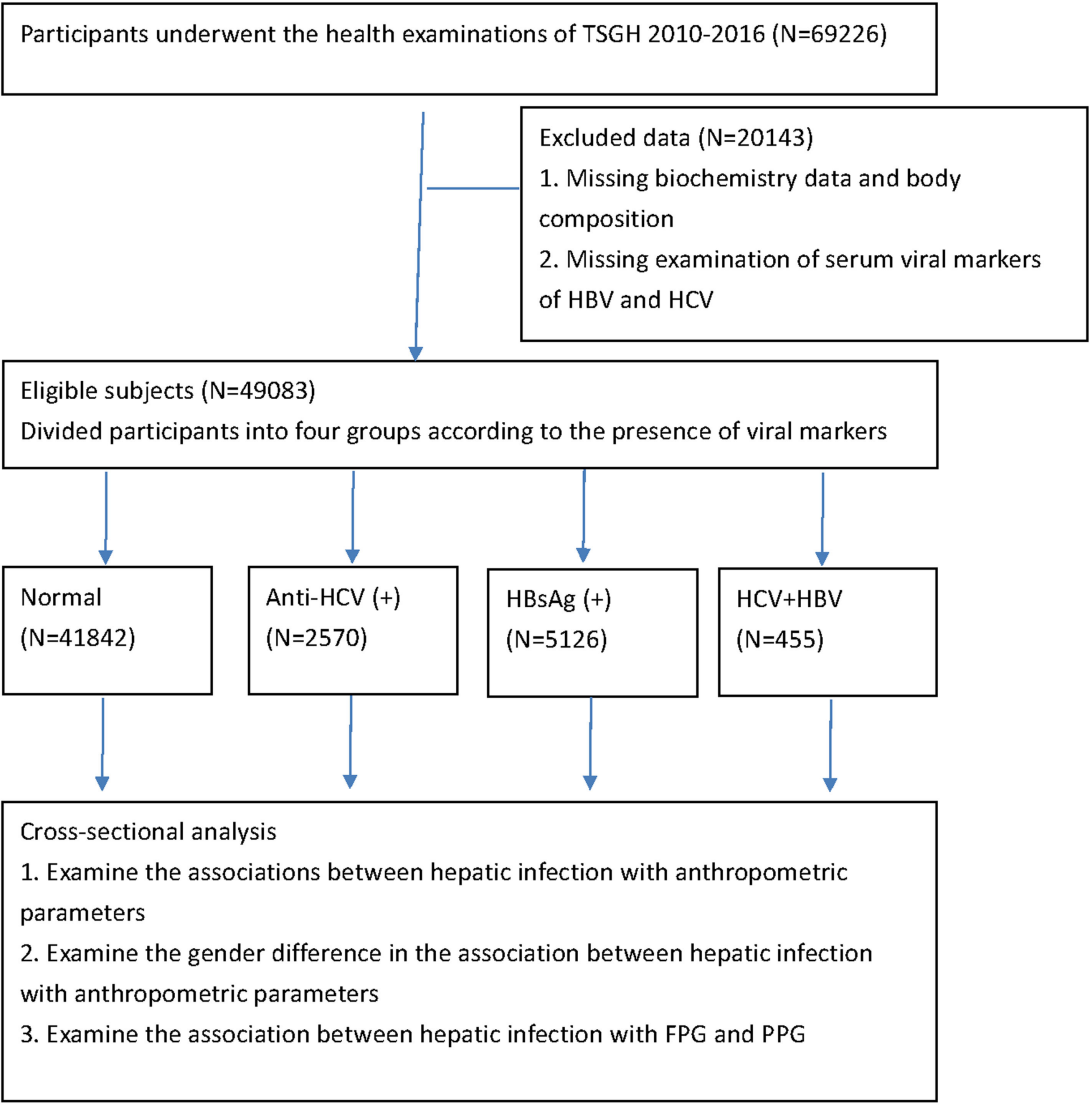
Flow chart of enrollment.

All analyzed patient characteristics were obtained from health examinations at Tri-Service General Hospital (TSGH) from 2010 to 2016, including 69226 participants aged 20 years and older. The study design met the requirements of the Helsinki Declaration and the design was approved by the institutional review board of Tri-Service General Hospital. The institutional review board of Tri-Service General Hospital waived the need to obtain individual informed consent because the data were analyzed anonymously. Fig 1 presents the flow chart for each step of the analysis. First, 20143 subjects lacking biochemistry data and serum hepatitis viral markers, body composition measurements and self-reported medical histories were excluded. Next, 49083 eligible participants were classified into four groups based on the presence of HBsAg or anti-HCV: normal (HBsAg (-)/anti-HCV (-)), HBV (HBsAg (+)/anti-HCV (-)), HCV (HBsAg (-)/anti-HCV (+)), and HBV/HCV coinfection (HBsAg (+)/anti-HCV (+)). Then, the associations between hepatitis virus infections and anthropometric parameters were tested in a multivariable linear regression model and a cross-sectional analysis. Finally, the associations of hepatitis virus infections with fasting plasma glucose (FPG) and postprandial plasma glucose (PPG) were investigated.

### Examination of hepatitis B/C infection

Serum viral markers of hepatitis B surface antigen (HBsAg) and anti-HCV were tested during the health examinations at Tri-Service General Hospital. Radioimmunoassay kits (Abbott Laboratories, Chicago, IL, USA) were used for detecting HBsAg. Anti-HCV was detected by using a 3rd-generation enzyme immunoassay (Abbott Laboratories).

### Measurement of body composition

Body mass index (BMI) was used as an attempt to quantify the amount of tissue mass of an individual and as a standard for recording obesity[13]. BMI was estimated based on the formula of body mass divided by the square) of the height (kg/m2). Waist circumference (WC) was measured using the smallest circumference between the lower ribs and iliac crests. Bioelectrical impedance analysis (BIA) is an effective, validated method for assessing body composition[14]. It is an alternative to more invasive and expensive methods such as dual-energy X-ray absorptiometry, computerized tomography, or magnetic resonance imaging. In the present study, we measured PBF and total lean mass (TLM) using BIA (InBody720, Biospace, Inc., Cerritos, CA, USA).

### Covariates measurement

Blood samples collected from subjects after fasting for at least 8 hours were used to analyze biochemical laboratory data. FPG and PPG were measured using a glucose oxidase method. Total cholesterol was measured by using an enzymatic colorimetric method. Other biochemical parameters were measured by standard laboratory tests.

### Statistical analysis

The Statistical Package for the Social Sciences, version18.0 (SPSS Inc., Chicago, IL, USA) for Windows was used for statistical calculations. The differences between the various hepatitis infection subgroups in terms of demographic information and biochemical data were tested with the Student’s t test and Pearson's chi-square test. The threshold for statistical significance was defined by a two-sided *p*-value of ≤ 0.05. The extend-model approach was applied for multivariable adjustment for pertinent clinical variables. A multivariable linear regression model was applied for associations between the various hepatitis infection subgroups and anthropometric parameters and for the relationships between different hepatitis infection subgroups with FPG and PPG.

## Results

### Demographic characteristic of the study population

The characteristics of the study sample including age, body composition, laboratory data and medical history are summarized in Table 1. The mean ages of the various subgroups were as follows: Normal: 39.57±13.74 years; HCV: 44.18±13.73 years; HBV: 43.17±11.59 years; HCV/HBV: 46.02±11.29 years. The HCV/HBV coinfection group had higher levels of FPG (97.17±38.92 mg/dL), PPG (156.30±61.59 mg/dL), and prevalence of T2DM (6.4%) than other subgroups. Participants in the HCV group had higher PBF (28.86±7.25%) than did the other subgroups. All characteristic data showed significant differences, except serum for total cholesterol, uric acid and history of smoking.

**Table 1:** Characteristics of study sample.

Variables	Normal	HCV	HBV	HCV+HBV	P Value
**Continuous Variables, mean (SD)**					
**Age (years)**	39.57 (13.74)	44.18 (13.73)	43.17 (11.59)	46.02 (11.29)	<0.001
**Body mass index (kg/m**^**2**^**)**	23.87 (4.07)	24.00 (3.97)	24.21 (4.04)	23.76 (4.01)	<0.001
**Percentage body fat (%)**	28.23 (7.34)	28.86 (7.25)	27.96 (7.42)	28.33 (7.07)	0.045
**Total cholesterol (mg/dL)**	185.00 (35.59)	185.12 (36.60)	185.33 (33.39)	185.93 (35.65)	0.862
**FPG (mg/dL)**	92.68 (21.34)	93.53 (20.51)	94.31 (23.39)	97.17 (38.92)	<0.001
**PPG (mg/dL)**	142.28 (51.09)	146.67 (51.31)	138.53 (48.81)	156.30 (61.59)	0.020
**Uric acid (mg/dL)**	5.54 (1.48)	5.57 (1.47)	5.59 (1.44)	5.57 (1.48)	0.359
**Creatinine**	0.81 (0.31)	0.83 (0.48)	0.83 (0.31)	0.81 (0.26)	<0.001
**AST (U/L)**	20.15 (12.06)	20.83 (10.27)	24.97 (29.71)	22.36 (12.13)	<0.001
**Albumin (g/dL)**	4.52 (0.29)	4.46 (0.27)	4.51 (0.30)	4,43 (0.28)	<0.001
**HsCRP (mg/dL)**	0.24 (0.53)	0.26 (0.50)	0.19 (0.31)	0.17 (0.20)	0.007
**Category Variables, (%)**					
**Proteinuria**	11820 (32.1)	556 (30.8)	1263 (29.7)	141 (33.3)	0.006
**Pre-DM**	4856 (11.6)	290 (13.7)	615 (13.2)	48 (10.5)	0.002
**Type 2 DM**	1481 (3.6)	85 (4.2)	196 (4.3)	24 (6.4)	<0.001
**Smoking**	4017 (28.2)	329 (30.6)	501 (28.3)	58 (29.0)	0.570
**Drinking**	5936 (48.4)	491 (46.2)	647 (45.4)	86 (44.1)	0.010

### Association between hepatitis virus infections and anthropometric parameters

The associations of hepatitis virus infections and PBF, WC and TLM were analyzed by multivariable linear regression model (Table 2). HCV, HBV and HCV/HBV coinfection had positive association with PBF, with **β** values of 0.578, 0.425 and 0.997 (95%CI = 0.215, 0.941; 0.094, 0.756; 0.175, 1.820), respectively, in the fully adjusted model. In particular, subjects with HCV/HBV coinfection were more closely associated with PBF than were other subgroups. However, no interaction was noted among the hepatitis virus infections groups with WC and TLM.

**Table 2:** Association between hepatic infection and anthropometric parameters.

Variables	Model ^a^ 1 β^b^ (95% CI)	*P* Value	Model ^a^ 2 β^b^ (95% CI)	*P* Value	Model ^a^ 3 β^b^ (95% CI)	*P* Value
***PBF***						
**HCV**	0.617 (0.249, 0.984)	<0.001	0.580 (0.217, 0.944)	0.002	0.578 (0.215, 0.941)	0.002
**HBV**	0.399 (0.066, 0.732)	0.019	0.442 (0.111, 0.773)	0.009	0.425 (0.094, 0.756)	0.012
**HCV+HBV**	1.072 (0.240, 1.905)	0.012	0.984 (0.161, 1.807)	0.019	0.997 (0.175, 1.820)	0.018
***WC***						
**HCV**	0.310 (-0.183, 0.803)	0.217	0.299 (-0.193, 0.792)	0.233	0.306 (-0.186, 0.798)	0.223
**HBV**	0.262 (-0.195, 0.718)	0.261	0.289 (-0.169, 0.747)	0.217	0.307 (-0.151, 0.765)	0.189
**HCV+HBV**	0.964 (-0.136, 2.063)	0.086	0.993 (-0.105, 2.092)	0.076	0.966 (-0.132, 2.063)	0.085
***TLM***						
**HCV**	-0.247 (-0.542, 0.049)	0.102	-0.239 (-0.533, 0.055)	0.111	-0.237 (-0.531, 0.056)	0.113
**HBV**	-0.157 (-0.424, 0.111)	0.251	-0.151 (-0.418, 0.117)	0.271	-0.136 (-0.404, 0.131)	0.318
**HCV+HBV**	-0.513 (-1.182, 0.157)	0.133	-0.509 (-1.176, 0.157)	0.134	-0.520 (-1.185, 0.146)	0.126

^a^ Adjusted covariates:

Model 1 = age + gender + BMI

Model 2 = Model 1 + proteinuria, serum total cholesterol, uric acid, creatinine, AST, albumin, hsCRP

Model 3 = Model 2 + history of smoking, drinking

^b^ β coefficients was interpreted as change of PBF, WC, and TLM for each increase in different hepatic infection

A gender difference in associations between hepatitis virus infections and anthropometric parameters are displayed in Table 3. The previous results remained in the male population with **β** values of 0.828, 0.464 and 1.445 (95%CI = 0.337, 1.318; 0.033, 0.895; 0.314, 2.577), respectively, but not in the female population.

**Table 3:** Association between hepatic infection and anthropometric parameters in gender difference.

Gender	Variables	Model ^a^ 1 β^b^ (95% CI)	*P* Value	Model ^a^ 2 β^b^ (95% CI)	*P* Value	Model ^a^ 3 β^b^ (95% CI)	*P* Value
	***PBF***						
**Male**	**HCV**	0.845 (0.347, 1.343)	<0.001	0.813 (0.321, 1.305)	<0.001	0.828 (0.337, 1.318)	<0.001
	**HBV**	0.430 (-0.005, 0.866)	0.053	0.488 (0.056, 0.920)	0.027	0.464 (0.033, 0.895)	0.035
	**HCV+HBV**	1.596 (0.448, 2.744)	0.006	1.453 (0.319, 2.588)	0.012	1.445 (0.314, 2.577)	0.012
**Female**	**HCV**	0.329 (-0.214, 0.873)	0.235	0.264 (-0.271, 0.800)	0.333	0.270 (-0.266, 0.806)	0.323
	**HBV**	0.412 (-0.106, 0.930)	0.119	0.490 (-0.024, 1.003)	0.062	0.493 (-0.022, 1.007)	0.060
	**HCV+HBV**	0.459 (0.744. 1.662)	0.454	0.364 (-0.820, 1.543)	0.546	0.371 (-0.814, 1.557)	0.539
	***WC***						
**Male**	**HCV**	0.413 (-0.181, 1.006)	0.173	0.413 (-0.180, 1.005)	0.172	0.416 (-0.176, 1.008)	0.168
	**HBV**	0.133 (-0.396, 0.661)	0.622	0.113 (-0.417, 0.643)	0.676	0.125 (-0.405, 0.655)	0.644
	**HCV+HBV**	1.437 (0.042, 2.832)	0.044	1.479 (0.085, 2.874)	0.038	1.438 (0.044, 2.832)	0.043
**Female**	**HCV**	0.163 (-0.659, 0.984)	0.698	0.136 (-0.685, 0.956)	0.746	0.164 (-0.657, 0.985)	0.695
	**HBV**	0.301 (-0.505, 1.106)	0.465	0.426 (-0.382, 1.235)	0.301	0.461 (-0.348, 1.269)	0.264
	**HCV+HBV**	0.430 (-1.299, 2.158)	0.626	0.392 (-1.131, 2.115)	0.656	0.372 (-1.349, 2.092)	0.672
	***TLM***						
**Male**	**HCV**	-0.283 (-0.739, 0.172)	0.223	-0.282 (-0.736, 0.171)	0.222	-0.291 (-0.744, 0.162)	0.208
	**HBV**	-0.205 (-0.603, 0.193)	0.313	-0.207 (-0.606, 0.191)	0.308	-0.193 (-0.592, 0.205)	0.341
	**HCV+HBV**	-0.892 (-1.943, 0.159)	0.096	-0.830 (-1.877, 0.216)	0.120	-0.832 (-1.877, 0.214)	0.119
**Female**	**HCV**	-0.225 (-0.541, 0.091)	0.163	-0.197 (-0.512, 0.118)	0.219	-0.183 (-0.497, 0.132)	0.255
	**HBV**	-0.238 (-0.539, 0.064)	0.123	-0.229 (-0.531, 0.073)	0.137	-0.215 (-0.517, 0.087)	0.163
	**HCV+HBV**	-0.098 (-0.798, 0.603)	0.785	-0.086 (-0.782, 0.610)	0.809	-0.103 (-0.798, 0.593)	0.773

^a^ Adjusted covariates:

Model 1 = age + BMI

Model 2 = Model 1 + proteinuria, serum total cholesterol, uric acid, creatinine, AST, albumin, hsCRP

Model 3 = Model 2 + history of smoking, drinking

^b^ β coefficients was interpreted as change of PBF, WC, and TLM for each increase in different hepatic infection

### Association of hepatitis virus infections with FPG and PPG

A linear regression model was applied to the association of hepatitis virus infections with FPG and PPG (Table 4). Only HCV/HBV coinfection was closely associated with FPG and PPG, as opposed to the other subgroups after multivariable adjustment with **β** values of 12.063 and 15.906 (95%CI = 7.110, 17.016; 2.875, 28.937), respectively.

**Table 4:** Association between hepatic infection and FPG and PPG.

Variables	Model ^a^ 1 β^b^ (95% CI)	*P* Value	Model ^a^ 2 β^b^ (95% CI)	*P* Value	Model ^a^ 3 β^b^ (95% CI)	*P* Value
***Fasting plasma glucose***						
**HCV**	0.896 (-1.335, 3.126)	0.431	0.848 (-1.374, 3.069)	0.455	0.815 (-1.405, 3.035)	0.472
**HBV**	-0.786 (-2.852, 1.279)	0.455	-0.997 (-3.066, 1.071)	0.344	-0.991 (-3.058, 1.075)	0.347
**HCV+HBV**	12.075 (7.100, 17.051)	<0.001	12.278 (7.322, 17.235)	<0.001	12.063 (7.110, 17.016)	<0.001
***Postprandial plasma glucose***						
**HCV**	0.919 (-4.852, 6.690)	0.755	0.884 (-4.829, 6.596)	0.762	0.784 (-4.925, 6.493)	0.788
**HBV**	-3.230 (-8,164, 1.704)	0.199	-4.044 (-8.970, 0.882)	0.108	-4.277 (-9.202, 0.648)	0.089
**HCV+HBV**	16.457 (3.291, 29.624)	0.014	15.584 (2.545, 28.623)	0.019	15.906 (2.875, 28.937)	0.017

^a^ Adjusted covariates:

Model 1 = age + gender + BMI

Model 2 = Model 1 + proteinuria, serum total cholesterol, uric acid, creatinine, AST, albumin, hsCRP

Model 3 = Model 2 + history of smoking, drinking

^b^ β coefficients was interpreted as change of fasting plasma glucose and postprandial plasma glucose for each increase in different hepatic infection

## Discussion

The present study highlighted the important role of the interaction of hepatitis virus infection with obesity. Hepatitis virus infections such as HCV, HBV and HCV/HBV coinfection had a positive relationship with increased PBF, especially in males. HCV/HBV coinfection was significantly associated with increased FPG and PPG. This suggested that hepatitis virus infection might influence the mechanisms of glucose uptake and might cause the development of adiposity accumulation. To the best of our knowledge, our study was the first to explore the association between hepatitis virus infection and adipose tissue by estimating anthropometric parameters in a population-based cross-sectional study.

Previous studies examined the close relationship between liver diseases and glucose metabolisms. Interventions for DM such as pioglitazone, glucagon like peptide-1 (GLP-1), and sodium‐glucose cotransporter 2 inhibitor (SGLT2I) have been reported to provide benefits in nonalcoholic steatohepatitis[15–17]. Allison et al. first reported that patients with HCV-related cirrhosis had more T2DM than did those with cirrhosis from other etiologies[6]. Accumulated clinical and experimental studies demonstrated that HCV directly contributed to perturbed glucose metabolism, leading to both insulin resistance and diabetes[18, 19]. Glucose intolerance was reported to be prevalent in patients with HBV[20]. In a large population-based study, chronic HBV was associated with insulin resistance defined by HOMA-IR. Papatheodoridis et al. observed that diabetes was present in more than 10% of patients diagnosed with hepatitis virus infection without a significant difference between those with chronic hepatitis B or C[12]. The exact mechanisms of HCV-induced insulin resistance were not determined, but numerous potential viewpoints had been reported[21]. Impaired expression of insulin receptor substrates was noted among patients with HCV[22]. Impaired insulin resistance signaling pathways caused by HCV were included downregulation of PPARγ, activation of the mTOR/S6K1 pathway, and increased secretion of TNF-α[23–25]. HCV infection also promoted the expression of gluconeogenic genes resulting in glucose metabolic dysfunction, such as reduced glucose uptake and increased plasma glucose[26].

Several studies have found a higher prevalence of glucose metabolism disturbances in patients with HCV infection[27, 28]. Specifically, an increased risk for T2DM was noted in patients with chronic HCV infection[29]. T2DM occurred in 14.5–33.0% of chronic HCV patients[30, 31]. However, only 3.7% of participants were diagnosed with T2DM in the present study. As seen in Table 4, HCV infections were not associated with glucose abnormalities. One possible explanation for this was that all participants’ FPG levels were within normal range, and a low prevalence of T2DM was found. Neverthelss, higher levels of FPG and PPG were observed in HCV subjects than in the normal group. In addition, a higher prevalence of T2DM was observed in the HCV group than in the normal group. Notably, HCV/HBV coinfection was closely associated with increased FPG and PPG in a relatively healthy population, but there was no such association with HCV or HBV infection alone.

Insulin resistance was significantly related to adipose tissue, especially visceral adiposity[32]. Eguchi et al. suggested that HCV infection potentially influenced glucose metabolism regardless of the amount of visceral fat accumulation. Increasing visceral fat accumulation significantly developed insulin resistance in HCV-infected patients[33]. Specific cytokines produced by visceral adipose tissue, such as leptin, adiponectin and inflammatory factors including TNF-*α* and IL-6, might lead to insulin resistance[34]. This was consistent with our findings that hepatitis virus infections were related to increased PBF. The mechanisms of the interaction of body fat accumulation with HCV and HBV remained unknown and should be investigated in further studies.

Gender difference were noted in the relationship between hepatitis virus infections and PBF. Epidemiological studies revealed that chronic HBV and HCV infections progressed more rapidly in males than in females[35, 36]. The predominant liver diseases tending to occur in men and postmenopausal women were cirrhosis, nonalcoholic fatty liver disease (NAFLD) and hepatocellular carcinoma (HCC)[37]. In a previous study, estradiol was suggested to have a beneficial effect on the progression of chronic liver disease by suppressing hepatic fibrosis and reducing hepatocyte apoptosis[38].

Although the large population-based sample analyzed in our study was a strength, there are nevertheless several limitations. First, measurement of insulin resistance was absent in the health examinations. This measurement might be more accurate to test the association of glucose metabolism with hepatitis virus infection. Second, our data did not support a causal inference that might be drawn from a longitudinal survey, therefore such examination in the future is needed. Third, a causative effect of hepatitis virus infection on PBF was not established in the present study. Finally, we could not distinguish participants with spontaneously or therapeutically resolved HCV infection in this cross-sectional study based on past and medication histories. Serum anti-HCV may persist over time, decrease slightly, or disappear gradually after a few years in patients with spontaneously or therapeutically resolved infection[39]. The measurement of anti-HCV was only performed once in our study rather than repeated several times during a long-term follow-up. The diagnosis of chronic inactive HBsAg carrier status was made based on the absence of HBeAg, repeated normal liver function, and even normal histology on biopsy[40]. However, the examination of HBeAg or liver biopsy was unavailable in the current study, such that we could not determine the difference between inactive carriers and chronic hepatitis patients. As seen in Table 1, the majority of participants with hepatitis B infection had relatively normal liver function (AST: 24.97 U/L). It is tempting to speculate that most of our participants were chronic inactive carriers, not chronic hepatitis patients.

## Conclusion

Hepatitis virus infections including HCV, HBV and HCV/HBV coinfection were observed to be closely associated with increased PBF in male patients. The plausible underlying pathophysiologic mechanism concerning these associations might be related to increased glycemic status. Better recognition of the interactions between body composition and hepatitis virus infections might provide useful data for treatments applied in obesity-related diseases.
